# Effectiveness of Acupuncturing at the Sphenopalatine Ganglion Acupoint Alone for Treatment of Allergic Rhinitis: A Systematic Review and Meta-Analysis

**DOI:** 10.1155/2019/6478102

**Published:** 2019-03-12

**Authors:** Qinwei Fu, Lanzhi Zhang, Yang Liu, Xinrong Li, Yepeng Yang, Menglin Dai, Qinxiu Zhang

**Affiliations:** ^1^School of Acupuncture and Moxibustion and Tuina/Third Affiliated Hospital, Chengdu University of Traditional Chinese Medicine, Chengdu 610072, China; ^2^Teaching Hospital of Chengdu University of Traditional Chinese Medicine, Chengdu 610072, China; ^3^School of Medical and Life Sciences, Chengdu University of Traditional Chinese Medicine, Chengdu 610072, China

## Abstract

**Aim(s):**

To evaluate the efficiency of acupuncturing at the sphenopalatine ganglion acupoint alone for treatment of allergic rhinitis.

**Design:**

A total of ten online databases were searched to find studies published up to Jan. 2018. Primary outcome measures include the TNSS, the RQLQ score, the VAS score, total effective rate, score for signs and symptoms, and the improvement of disease classification. Study quality of each included article was evaluated by the Cochrane Collaboration risk of bias tool. A meta-analysis was conducted based on the Cochrane systematic review method by using RevMan 5.3 software.

**Interventions:**

Acupuncturing SGA alone was the only therapy in experimental group. Interventions in control groups includes sham acupuncture, acupuncturing other regular acupoints, and western medicine. Specific techniques included manual acupuncture and electroacupuncture only.

**Primary Outcomes:**

They include TNSS, RQLQ, VAS score, total effective rate, the improvement of disease classification.

**Results:**

Ten studies of eight articles involving 1004 participants were included. Result of meta-analysis showed that acupuncturing sphenopalatine ganglion acupoints alone was more effective than control groups. However, several adverse effects were reported.

**Conclusion:**

These findings show that acupuncturing the sphenopalatine ganglion acupoint alone has a potential role in alleviating nasal symptoms, improving quality of life for patients, and the effectiveness of acupuncture in the treatment of allergic rhinitis, suggesting it as a considerable therapy for allergic rhinitis. However, more studies are needed to execute a subgroup analysis of various variables and to evaluate the publication bias of the study.

## 1. Introduction

Allergic rhinitis (AR), a nasal symptomatic disorder induced after allergen exposure, is characterized by nasal congestion and discharge, sneezing, and nasal itching [1], and it is estimated to affect about 1.4 billion people globally and continues to be on the rise [2]. AR causes major illness and disability worldwide and reduces the quality of life and productivity regardless of ethnicity, gender, and age [3].

Conventional medical treatments for AR include H1-antihistamines, leukotriene antagonists, glucocorticosteroids, anticholinergics, decongestants, and specific immunotherapy [4]. In spite of the clinical effects of these conventional treatments, their adverse reactions cause concern. Treatment combining complementary and alternative medicine improves clinical effects and reduces the incidence of adverse reactions [5]. As a result, an increasing number of patients seek complementary and alternative treatments [5, 6].

However, because of the lack of sufficient experimental and clinical evidence supporting its efficacy, acupuncture was not widely recommended for the treatment of AR in the past [4]. From 2015, increasing evidence especially from multicenter randomized controlled clinical trials has proven the efficiency of acupuncture in treating AR [7–10]. Therefore, acupuncture is listed on the AR Clinical Guidelines in USA now [11].

The SGA is located in the double cheek [12, 13] and near ST7 (*Xiaguan*) [14]. Technically, the needle is inserted through SGA to reach the pterygopalatine fossa [15] to stimulate the sphenopalatine ganglion (SG) and other nasal nerves [16], increasing nasal ventilation and decreasing glandular secretion. The use of acupuncturing at the SGA to treat AR was firstly reported in 1990 [13].

The curative effect of acupuncture on AR is mediated by anti-inflammatory effects through the regulation of the interaction between the vagus nerve and macrophages, which directly prevent the development of AR [17]. In traditional Chinese medicine, physicians have used acupuncture at other regular acupoints to treat allergic rhinitis for many years effectively [18, 19]. In 2011, a large clinical study suggested that acupuncturing at the sphenopalatine ganglion acupoint, a technique developed by a Chinese otolaryngologist and applied in more than 130,000 Chinese patients [20], offers potential advantages with regard to nasal symptoms, onset time, duration of effectiveness, and quality of life. However, it has been limited by the lack of evidence in evidence-based medicine to evaluate the efficiency of acupuncturing SGA alone in the treatment for AR.

The aim of this systematic review and meta-analysis is to determine the effectiveness of acupuncturing at the SGA for treating AR and to compare the efficacy and adverse effects of sham acupuncture, acupuncturing at ORA, and WM by several comparisons. We provide further evidence of quantity and quality in order to draw more definitive conclusions. In this systematic review and meta-analysis, we further evaluated the total effective rate, RQLQ score, TNSS and TNNSS score, and rhinitis symptom scale of acupuncturing SGA alone in the treatment of allergic rhinitis.

## 2. Materials and Methods

### 2.1. Protocol and Registration

This systematic review was registered in PROSPERO, an international prospective register of systematic reviews, with the registration number CRD42018107322 (available from https://www.crd.york.ac.uk/prospero/display_record.php?RecordID=107322).

### 2.2. Search Strategy

The search strategy was decided according to the guidance of the PRISMA agreement (http://www.prisma-statement.org/). The electronic databases that we searched include PubMed, Embase, MEDLINE, Springer, Proquest, Cochrane Library, the China National Knowledge Infrastructure (CNKI), Chinese Science and Technology Periodical Database (VIP) SinoMed (CBM), and Wanfang Data Information Site. The articles included are published from the inception of each database up to August 2018. The languages were limited to English and Chinese. There are two groups of search terms: intervention (acupuncture, acupuncturing, stimulus, stimulate, sphenopalatine ganglion, ganglia pterygopalatinum, Merkel's ganglion, and other related terms in Chinese) and object (allergic rhinitis, AR, rhinallergosis, and other related terms in Chinese). All searches were limited to studies of RCT in humans and were conducted in electronic databases by two authors independently. We also tried to get grey literatures identified through other sources.

### 2.3. Inclusion Criteria

Studies were included if the following criteria were met:

(1) Subjects: studies on patients with AR treated by acupuncturing at SGA and parallel-group, randomized clinical trials regardless of blinding or publication types.

(2) Interventions: acupuncturing SGA alone was the only therapy except for control groups. Specific techniques only included manual acupuncture and electroacupuncture.

(3) Primary outcomes: the primary outcomes include the TNSS, RQLQ, VAS score, total effective rate, and the improvement of disease classification.

(4) Additional outcomes: the additional outcomes include the TNNSS, relative laboratory index, for example, IgE, IL, recurrence rate, time when the symptoms disappear, frequency of seizure, and the case of drug reduction.

(5) Language: Chinese and English.

(6) Participants: participants with all types of AR (including intermittent and persistent) regardless of age, gender, etiology, severity, and ethnic group, and diagnosed with specific criteria (i.e., mention any one of the criteria for diagnosis of AR) were eligible for inclusion.

### 2.4. Exclusion Criteria

Studies were excluded if the following criteria were met: (1) moxibustion, auricular acupuncture, scalp acupuncture, and other forms of acupuncture; (2) studies in which Chinese herbal medicine or WM were used as intervention measures of experimental groups; (3) studies that compared different acupuncture techniques or different acupoints; (4) nonrandomized controlled trials; (5) duplicate publications; and (6) animal experiments, anatomy, and other basic studies.

### 2.5. Study Selection and Data Extraction

According to the design above, two reviewers (Dai ML and Fu QW) searched listed online databases and listed the titles and abstracts of all the articles. Two evaluators (Liu Y and Li XR) assessed the eligibility of these articles and made decisions on every research (inclusion or exclusion) independently. If they did not reach the same decision, the concerned articles were discussed with a third reviewer (Zhang QX). Two reviewers (Yang YP and Fu QW) extracted data independently from each study. Differences of extracted data were solved after discussion with a third reviewer (Zhang QX).

### 2.6. Quality Assessment

Quality assessment of all the studies included in this review was independently evaluated by two reviewers (Fu QW and Yang YP) using the Cochrane Collaboration risk of bias tool by RevMan 5.3 software (2,3). Any disagreement was resolved by discussion with the third author (Zhang QX).

### 2.7. Statistical Analysis

The meta-analysis was performed with the RevMan 5.3 software (3). Some results including the TNSS, TNNSS, and RQLQ score were considered as continuous variables and the total effective rate is considered as dichotomous data. Mean difference (MD) and risk ratio (RR) with 95% CIs were given separately, which was an estimate of the combined effect sizes, and* P* values of less than 0.05 were considered statistically significant.

For the assessment of heterogeneity, we evaluated studies using both the I^2^ statistic and the Chi-square test (*p < *0.1), which indicates the proportion of variability across studies not explained by sampling variation alone, and the* p* value of the V^2^ test of heterogeneity.

If multiple studies existed, they would be subject to a meta-analysis and if the studies were too heterogeneous to conduct a meta-analysis, then the evidence for noninferiority, equivalence, or superiority would be assessed for consistency across the studies.

For pooled data, we used a random-effects model for these comparisons.

Exploration of publication bias was planned if more than ten studies were included. Due to the number of included studies and methodological quality, not all planned analyses could be available.

## 3. Results

### 3.1. Study Inclusion

**Figure 1:**
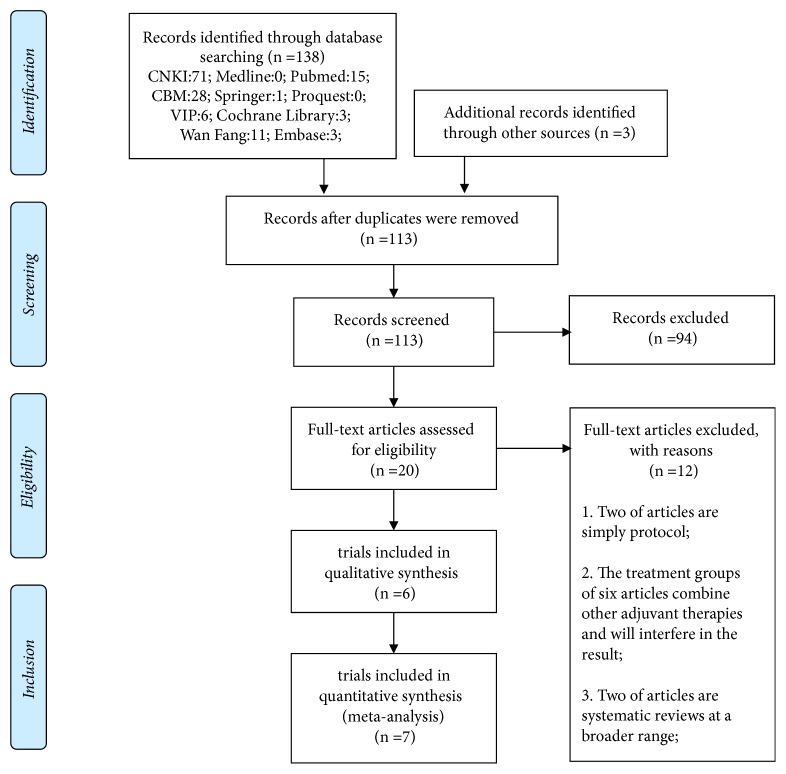
Flowchart of study selection.

Initially, 141 records were searched from ten databases with three grey literature references. After removing duplicates, the records were decreased to 113. Based on titles and abstracts of records, we excluded 94 papers with reasons, if they were an animal experiment, case report or review, not related to acupuncturing SGA, and so on. In the next, due to republications, nonrandomized controlled trials, not studies on patients with AR treated by acupuncturing SGA, and so on, 86 articles were excluded. The 20 remaining articles were downloaded for further selection. Eventually, ten studies of eight trials from eight articles were included [10, 21–27]. The flow diagram of the study selection process is shown in Figure 1.

### 3.2. Study Characteristics

Eight included trials were conducted and published in China, and only one was published in English [10], while others were all published in Chinese. In total, 1004 participants with AR were involved in ten studies, aged between 9 and 70, and the duration of disease varied from 5 months to 50 years. Two studies involved participants younger than 18 years [26, 27]. Detailed characteristics of the studies are listed in Table 1. Manual acupuncture and electroacupuncture were applied for the acupuncturing SGA intervention, and a variety of prescriptions such as acupuncturing ORA, sham acupuncture, and WM were applied for the control groups. Although some of the ORA in the control groups were different among the included studies, they are widely used according to the theory of traditional Chinese medicine. Thus, they were considered as the same orientation. Eight studies reported the participants with the total effective rate of treatment acupuncturing SGA (including significant effective rate and effective rate), which were applied for dichotomous data. Nine studies reported the scores of several types of scales of symptoms and signs (i.e., the TNSS, the TNNSS, the RQLQ, and the score for signs and symptoms), which were applied for continuous variables.

### 3.3. Description of Control Interventions

All of the included eight studies used some interventions in control groups (Table 1). Control interventions consisted of WM, sham acupuncture, and acupuncturing ORA. WM was adopted in two studies [21, 27], acupuncturing ORA was adopted in six studies [21–23, 26, 27], and two experiments used sham acupuncture [10, 24]. Control interventions were administered for similar treatment duration as acupuncturing SGA. Types of control medication consisted of budesonide nasal spray [21] and lynette capsule [27]. Shallow acupuncture was used as sham acupuncture in two experiments.

### 3.4. Assessment of Quality and Bias

**Figure 2:**
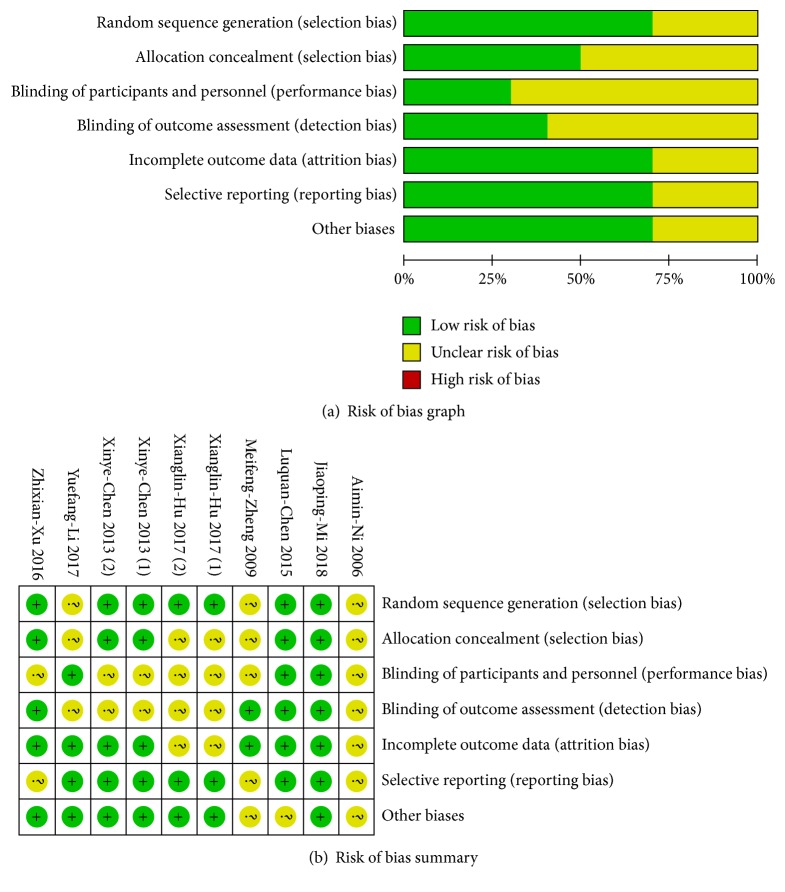
Risk of bias graph and summary.

According to the results of Cochrane Collaboration risk of bias tool, nine of the ten studies in total were of unclear risk of bias. The method of randomization was described clearly and was appropriate in seven studies [10, 21, 22, 24, 25]; five of the studies described the method of allocation concealment clearly [10, 21, 22, 24]. Three were single-blinded to participants [10, 24, 26], four were single-blinded to outcome assessment [10, 22–24], and two were double-blinded [10, 24]. The bias for each study is shown below (Figure 2(a)), and the bias summary is shown in Figure 2(b).

### 3.5. Effectiveness of Acupuncture Sphenopalatine Ganglion Alone in AR Patients

#### 3.5.1. Acupuncturing Unilateral SGA Alone versus Acupuncturing ORA

**Figure 3:**
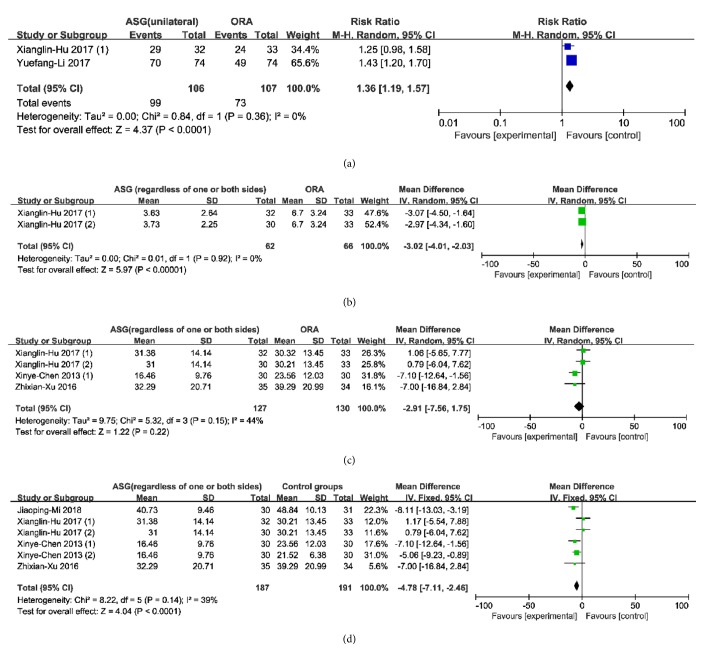
Forest plots of acupuncturing SGA versus others.

By comparing the total effective rate, a total of two studies showed that acupuncturing unilateral SGA alone was more effective than acupuncturing ORA [25, 26]. There was no heterogeneity of these two studies by comparing the total effective rate (heterogeneity: Chi^2^ = 0.84, df = 1 (*P* = 0.36); *I*^2^ = 0%) with the random-effects model (RR = 1.36, CI 1.19 to 1.57) used (Figure 3(a)). The results showed that acupuncturing unilateral SGA alone was more effective in treating AR than acupuncturing ORA.

#### 3.5.2. Acupuncturing SGA Regardless of One or Both Sides Alone versus Acupuncturing ORA

By comparing the total effective rate and RQLQ scores, a total of three studies showed that acupuncturing bilateral SGA alone was more effective than acupuncturing ORA [21, 23, 25]. There was a severe and unacceptable heterogeneity of these three studies [21, 23, 25] by comparing the total effective rate (heterogeneity: Chi^2^ = 14.69, df = 2 (*P* = 0.0006); *I*^2^ = 86%) with the random-effects model (RR = 1.28, CI 0.87 to 1.88) used. And a moderate but unacceptable heterogeneity of the included two of the three studies [21, 25] by comparing the RQLQ score (heterogeneity: Chi^2^ = 3.05, df = 1 (*P* = 0.08); *I*^2^ = 67%) with the random-effects model (MD = -3.43, CI -11.15 to 4.28) was used. After a sensitivity analysis was conducted in both comparisons, the heterogeneity was still severe and unacceptable. Subgroup analysis and meta-regression analysis are not applicable in both comparisons for they included fewer than ten studies, so we removed the two comparisons at last.

#### 3.5.3. Acupuncturing Bilateral SGA Alone versus WM

By comparing the total effective rate, a total of two studies showed that acupuncturing bilateral SGA alone was more effective than WM [21, 27]. There was a severe and unacceptable heterogeneity of these two studies by comparing the total effective rate (heterogeneity: Chi^2^ = 14.05, df = 1 (*P* = 0.0002); *I*^2^ = 93%) with the random-effects model (RR = 1.18, CI 0.88 to 1.57) used. After a sensitivity analysis was conducted, the heterogeneity was still severe and unacceptable. Subgroup analysis and meta-regression analysis are not applicable in this comparison for fewer than ten studies were included, so we removed this secondary comparison at last.

#### 3.5.4. Acupuncturing Bilateral SGA Alone versus Acupuncturing ORA

By comparing the total effective rate and RQLQ scores, a total of three studies showed that acupuncturing bilateral SGA alone was more effective than acupuncturing ORA [21, 23, 25]. There was a severe and unacceptable heterogeneity of these three studies [21, 23, 25] by comparing the total effective rate (heterogeneity: Chi^2^ = 14.69, df = 2 (*P* = 0.0006); *I*^2^ = 86%) with the random-effects model (RR = 1.28, CI 0.87 to 1.88) used. And a moderate but unacceptable heterogeneity of the included two of the three studies [21, 25] by comparing the RQLQ score (heterogeneity: Chi^2^ = 3.05, df = 1 (*P* = 0.08); *I*^2^ = 67%) with the random-effects model (MD = -3.43, CI -11.15 to 4.28) was used. After a sensitivity analysis was conducted in both comparisons, the heterogeneity was still severe and unacceptable. Subgroup analysis and meta-regression analysis are not applicable in both comparisons for they included fewer than ten studies, so we removed the two comparisons at last.

#### 3.5.5. Acupuncturing SGA Regardless of One or Both Sides Alone versus Acupuncturing ORA

By comparing the TNSS and TNNSS score, the RQLQ score, and the total effective rate, a total of seven studies showed that acupuncturing SGA of one or both sides alone was more effective than acupuncturing ORA [21–23, 25–27]. Firstly, there was no heterogeneity of the included two of seven studies [25] by comparing the TNSS and TNNSS score (heterogeneity: Chi^2^ = 0.01, df = 1 (*P* = 0.92); *I*^2^ = 0%) with the random-effects model (MD = -3.02, CI -4.01 to -2.03) (Figure 3(b)). Secondly, a mild and acceptable heterogeneity of the included four of seven studies [21, 22, 25] by comparing the RQLQ score (heterogeneity: Chi^2^ = 5.32, df = 3 (*P* = 0.15); *I*^2^ = 44%) with the random-effects model (MD = -2.91, CI -7.56 to 1.75) was used (Figure 3(c)). Thirdly, a severe and unacceptable heterogeneity of the included all studies [21–23, 25–27] by comparing the total effective rate (heterogeneity: Chi^2^ = 14.69, df = 2 (*P* = 0.0006); *I*^2^ = 86%) with the random-effects model (RR = 1.28, CI 0.87 to 1.88) was used. The first and the second results showed that acupuncturing SGA regardless of one or both sides alone was more effective in treating AR than that in traditional Chinese acupuncture. As for the last results, after a sensitivity analysis was conducted in both comparisons, the heterogeneity was still severe and unacceptable. Subgroup analysis and meta-regression analysis are not applicable in both comparisons for they included fewer than ten studies, so we removed both secondary comparisons at last.

#### 3.5.6. Acupuncturing SGA Regardless of One or Both Sides Alone versus Control Groups (Sham Acupuncture, WM, and Acupuncturing ORA)

By comparing the total effective rate, the TNSS, and the RQLQ, a total of 10 studies showed that acupuncturing SGA of one or both sides alone was more effective than control groups (sham acupuncturing, WM, and acupuncturing ORA) [10, 21–27]. Firstly, there was a severe and unacceptable heterogeneity of eight studies [21–23, 25–27] by comparing the total effective rate (heterogeneity: Chi^2^ = 44.96, df = 7 (*P* < 0.00001); *I*^2^ = 84%) with the random-effects model (RR = 1.17, CI 1.02 to 1.34). Secondly, there was a mild and acceptable heterogeneity of six studies [10, 21, 22, 25] by comparing the RQLQ (heterogeneity: Chi^2^ = 8.22, df = 5 (*P* = 0.14); *I*^2^ = 39%) with the random-effects model (MD = -4.51, CI -7.61 to -1.41) (Figure 3(d)). Thirdly, there was a severe and unacceptable heterogeneity of six studies [10, 21, 24, 25] by comparing the TNSS (heterogeneity: Chi^2^ = 60.32, df = 5 (*P* <0.00001); *I*^2^ = 92%) with the random-effects model (MD = -1.40, CI -2.38 to -0.42). The second result showed that acupuncturing SGA regardless of one or both sides alone was more effective in treating AR than sham acupuncture, WM, and acupuncturing ORA. As for the first and the third results, after a sensitivity analysis was conducted, the heterogeneity was still severe and unacceptable. Subgroup analysis and meta-regression analysis are not applicable for this comparison included fewer than ten studies, so we removed this secondary comparison at last.

### 3.6. Adverse Events Reported in Studies

Only three of eight trials mentioned adverse events in acupuncturing SGA groups [22, 24, 25].

Firstly, one patient reported an adverse event in acupuncture unilateral SGA alone group among 20 AR patients of the group and 39 of the study [22]. The adverse event in this group was hematoma occurring on the cheek after acupuncture, and the blood stasis was finally absorbed within three days.

Secondly, two patients reported adverse event in the group of acupuncturing SGA alone, not mentioning one or both sides among 40 AR patients of the group and 80 of the study [24]. The adverse events in this group were bleeding after acupuncture. After about three days, the adverse reactions disappeared.

Thirdly, one patient reported adverse event in acupuncturing unilateral SGA alone group among 32 AR patients of the group and 95 of the study [25]. The adverse event in this group was subcutaneous hematoma. After applying an ice compress, the patient's subcutaneous hematoma improved significantly within a week.

No adverse events were reported in the control groups of all the three studies.

## 4. Discussion

To our knowledge, this is the first systematic review and meta-analysis to evaluate the efficiency of acupuncturing SGA alone in the treatment for AR, and we could separate the pure effect of acupuncturing SGA alone treatment from other combined therapeutic interventions.

Ten studies involving 943 participants, with the sample sizes ranging from 20 to 386, were identified, and information available for the four comparisons was synthesized from five of them in this review [10, 21, 22, 25, 26]. This systematic review and meta-analysis found no strong enough conclusive evidence about the efficiency of acupuncturing SGA alone in patients with AR, which is likely a reflection of the limitation of the studies. However, our pooled results showed that acupuncturing SGA alone was superior to acupuncturing ORA, sham acupuncture, and WM in many aspects including the score of RQLQ, THSS and TNNSS, and the total effective rate.

The acupuncturing ORA therapy, WM therapy, or sham acupuncture was used in control groups including some commonly used acupoints of traditional Chinese acupuncture for AR such as Yintang (GV 29), Baihui (GV 20), yingxiang (LI20) Hegu (LI 4), Taichong (LR 3), Feishu (BL 13), and Dazhui (GV 14) and commonly used WM including tranilast capsules and budesonide nasal spray. The results showed that, compared with them, though not obvious from the existing evidence, acupuncturing SGA alone is better at decreasing symptoms of AR measured by the score of RQLQ, TNSS and TNNSS, and the total effective rate.

We adopted strict inclusion and exclusion criteria and applied a recognized tool to evaluate the quality of the included studies, and four comparisons were available for analysis.

All of the 10 studies in eight trials were finally included in four comparisons of meta-analysis with a low heterogeneity, and three of four comparisons favored that acupuncturing SGA is more effective compared with sham acupuncture, WM, and acupuncturing ORA therapy. Firstly, two studies [25, 26] reported that acupuncturing unilateral SGA alone was more effective than acupuncturing ORA measured by the total effective rate. Secondly, two studies [25] reported that acupuncturing SGA (regardless of one or both sides) alone was more effective than acupuncturing ORA measured by the TNSS and TNNSS. Thirdly, six studies [10, 21, 22, 25] reported that acupuncturing SGA (regardless of one or both sides) alone was more effective than acupuncturing ORA, sham acupuncture, and WM measured by the RQLQ score.

Based on this, we strongly suspect that acupuncturing SGA alone is noninferior to acupuncturing ORA and WM therapies in alleviating the symptoms of AR considering the side effect of WM therapy as well [6].

And, with the results of considerable efficiency of acupuncturing ORA for AR patients compared with sham/ placebo acupuncture or blank/wait control in control groups in previous trials [7–9], we strongly suspect that acupuncturing SGA is a kind of treatment with considerable efficiency for AR patients.

However, pooled data from three of the four studies showed that improvement of life quality is similar for treatments with acupuncturing ORA by comparing the RQLQ score. This result showed that acupuncturing SGA alone was not superior enough in treating AR.

For the outcome of adverse events, three studies reported number of events, possible triggers, symptoms, and treatment.

These ensured, to some extent, that our review could serve as an up-to-date and comprehensive summary of the published evidence on the topic of treatment for AR patients.

Given the different measures of outcomes adopted in the ten studies, it was difficult to combine and analyze the information in meta-analysis with a larger quantity. Though they can be assessed in the four smaller comparisons, more studies were needed to support the conclusion.

In terms of the treatment methods of the control groups, two literatures included in this study used western medicine. Chen (2013) used budesonide nasal spray (produced by AstraZeneca Pharmaceutical Co., LTD.) in one of the two control groups in a total of 30 patients with allergic rhinitis, 1 spray per side of the nostril, 64ug/spray, once in the morning and once in the evening, 2 weeks/course, a total of 2 courses, and did not use other drugs to treat allergic rhinitis during the treatment. By observing the effective rate (based on the VAS score of the four symptoms of sneezing, runny nose, nasal congestion, and nasal itching), at the end of the first course of treatment, the significant efficiency of acupuncture in sphenopalatine ganglion group and western medicine group was 50.7% and 48.6%, respectively, which was significantly better than that in the traditional acupuncture group (29.6%). After statistical treatment (P=0.021 and P=0.026), there was a significant difference. At the end of the second course of treatment, there was no significant difference in efficacy among the three groups (P=0.866); that is, the efficacy of sphenopalatine ganglion acupuncture group and traditional acupuncture group was similar to that of western medicine group. With the passage of time, the efficacy of sphenopalatine ganglion acupuncture group gradually decreased, but the efficacy of sphenopalatine ganglion acupuncture group was still higher than that of the traditional acupuncture group at 3 months and 6 months after treatment, with statistical difference (P=0.008 and P=0.001), which was the same as that of the western medicine group. In terms of RQLQ score comparison, after the completion of treatment, the quality of life of the patients in the three groups was improved due to the control of nasal symptoms, and the RQLQ score comparison before and after treatment was significantly reduced (P<0.01) and the sphenopalatine ganglion group and the traditional acupuncture group had no statistical significance compared with the western medicine group, respectively, while the latter had statistical significance (P<0.05), which indicated that all three treatments could reduce the RQLQ score of allergic rhinitis. The reduction range of the sphenopalatine ganglion group was better than the traditional acupuncture group, but it was unknown compared with the western medicine group. In the control group of 191 patients with allergic rhinitis, Ni (2006) used oral tranilast capsule, 0.2g/time, 3 times/day, 7 days a course of treatment, and did not use other drugs to treat allergic rhinitis during the treatment. The effective rate was observed (scored according to VAS score of clinical symptoms and nasal local signs score of four symptoms: sneezing, runny nose, nasal congestion, and nasal itching). Among them, in the sphenopalatine ganglion group (experimental group), 102/195 (52.33%) were significantly effective, 80/195 (41.03%) were effective, 13/195 (6.66%) were ineffective, and the total effective rate is 93.36%, In the oral western medicine group, 65/191 (34.03%) were significantly effective, 68/191 (35.60%) were effective, 57/191 (29.80%) were ineffective, and the total effective rate is 69.63%. There was significant difference between the two groups (P < 0.01). However, according to the existing literature, the heterogeneity of the two groups is high and cannot be solved. Therefore, according to the existing literature, we cannot make an effective comparison between needling sphenopalatine ganglion and taking western medicine.

In spite of this, the precise effects of acupuncturing SGA for treating AR remain uncertain given the significant overall risk of bias in our included studies. Thus, well-designed and large-sized RCTs are needed.

## 5. Limitations

Several limitations of this systematic review and meta-analysis should be mentioned below.

First, some of the negative results may not have been published and excluded; as a result, publishing bias is impossible. Failure to report details of design methodology is also a potential source of increased heterogeneity in the included studies.

Second, the funnel plots may be useful in investigating publishing bias but the number of the included studies in this review is no more than ten, so assessment of publication bias based on the funnel plots and meta-regressions cannot be carried out.

Third, seven of the eight trials were published in Chinese [21–27], and all of them were conducted in China, the country where acupuncture is well endowed, widely researched, and practiced. Further studies should be international and be conducted in multiple languages.

Fourth, the risk of bias of the included studies in this meta-analysis was not considerable in general, indicating that the quality was not high.

Fifth, only two in all the ten studies were performed with sham/placebo acupuncture or blank/wait control [10, 24]; it is impossible to evaluate the therapeutic effect in multiple dimensions.

Last, generally, nearly all the studies included focused on short-term outcomes (from 7 days to 24 weeks) only, and follow-up duration with long term was reported merely in one study. So, further prognosis cannot be determined without adequate information.

## 6. Conclusion

The current study indicates that, compared with WM, acupuncturing ORA, and sham acupuncture, acupuncturing SGA alone is an effective treatment for AR patients, especially in relieving nasal symptoms and improving quality of life. Moreover, it still needs more studies to execute a subgroup analysis of various variables due to the high heterogeneity of some studies and comparisons and to evaluate the publication bias of the study.

Because of these limitations, studies with rigorous designs, large samples, sham/placebo acupuncture or blank/wait control, and accurate reporting are needed in the future to enhance the power of evidence for the use of acupuncturing SGA in AR patients. Additionally, because all of the studies reviewed were conducted in China, further international studies outside of China are warranted to improve the applicability and generalizability of the results.

## Figures and Tables

**Table 1:** Characteristics of the included studies.

Study	Age		Course of the disease		Diagnosis	Intervention		Sample size (male/female)		Outcomes	Frequency (period)		Follow-up time	Withdrawal or lost to follow-up		Adverse events	Place
	(A/C)		(A/C)			(A/C)		A	C		(A/C)			(A/C)		(A/C)	

Chen 2015	21~39/21~39		More than 4 W/more than 4 W		A1	SGA/SA		11/9	9/10	A2, A9	1 per W (3W)/1 per W (3W)		NR	2/4		Blood stasis occurs after acupuncture on the cheek: 1/none	Beijing

Chen 2013 (A) *∗*	21~58 (37.0 ± 10.3)	ORA:24~53 (36.7 ± 8.7)	55.6M (mean)	53.4M (mean)	A3	SGA with EA	ORA with EA	13/17	15/15	A4, A9, A13, A14, A15	2 per W (4W)	2 per W (4W)	NR	NR		NR	Guangzhou
Chen 2013 (B) *∗*		WM:19~55 (35.0 ± 11.2)		46.7M (mean)			WM		14/16			2 per D (28D)					

Xu 2016	39 ± 10/42 ± 10		61.2 ± 49.2M/59.7 ± 58.2M		A5, A6	SGA/ORA		15/25	12/28	The evaluation of the curative effect according to symptoms and signs of A3, A4, A7, A8, A13	1 or 2 per W (1M, 8 at most)/2 per W (1M, 8 in total)		NR	5/6		Bleeding after acupuncture: 2/none	Beijing

Ni 2006	9~58 (mean=31.3)/11~61 (mean=33.5)		2~15Y (mean=5.5Y)/1.5~16Y (mean=4.5Y)		NR	SGA/WM		106/89	103/88	The evaluation of the curative effect according to symptoms and signs of A3, A13	1 per D (7D)/3 per D (7D)		NR	None		NR	Huzhou

Li 2017	29.6 ± 13.4/27.1 ± 17.2		11.5 ± 6.9Y/10.6 ± 7.2Y		NR	SGA/ORA		48/26	74/45	A13	1 per W (2M)/1 per D (2M)		NR	NR		NR	NR

Zheng 2009	NR		NR		A3	SGA/ORA		8/12	Healthy people: 9/11	(A9+A10); serum SP content; A13	1 per 2D for 30 times, take a 7-day break every two times (both groups)		Y	None/2		NR	Fuzhou
									ORA: 8/12								

Hu 2017 (A) *∗*	SGA (unilateral): 19~66 (49.22 ± 14.05)	18~69 (49.91 ± 10.61)	5M~32Y (12.36 ± 10.15Y)	6~50Y (12.77 ± 12.02Y)	A4	SGA (bilateral and unilateral separately)/ORA		13/19	16/17	A4, A9, A10, A13	1 per W (4W)	2 per W(4W)	NR	3	2	Subcutaneous hematoma (only 1 in unilateral SGA group)	Beijing
Hu 2017 (B) *∗*	SGA (bilateral): 18~70 (49.17 ± 13.86)		6M~30Y (10.82 ± 8.42Y)					12/18			1 per W (4W)			5			

Mi 2018	35.87 ± 10.36/35.97 ± 9.15		49.03 ± 29.84M/51.17 ± 32.39M		A6	SGA/SA		16/14	17/14	A4, A9, A14, A15	2 per W (4W)/2 per W (4W)		Y	6/5		None	Guangzhou

*∗*: Chen 2013 (A) and Chen 2013 (B) are the two substudies of Chen 2013, and Hu 2017 (A) and Hu 2017 (B) are the two substudies of Hu 2017; A1: Diagnostic Criteria for Diagnosis and Therapeutic Evaluation of Allergic Rhinitis (revised in 1997, Haikou); A2: Subjective Symptom Scale and Objective Evaluation Indexes of Patients with Rhinitis (Nasal resistance, Nasal acoustic reflex, Nasal exhalation NO); A3: Principles and Recommendations for the Diagnosis and Treatment of Allergic Rhinitis (Lanzhou, 2004); A4: the RQLQ Questionnaire; A5: Guidelines for the Diagnosis and Treatment of Allergic Rhinitis (2009, Wuyi mountain); A6: Allergic Rhinitis and Its Impact on Asthma (ARIA, 2008, WHO); A7: Visual Analogue Scale (VAS); A8: Follow-up on the number of days of recurrence; A9: Rhinitis Symptom Score (TNSS); A10: Score of the Accompanying Symptoms of Rhinitis (TNNSS); A11: Duration of efficacy after treatment; A12: the time required for improvement of symptoms, signs, and accompanying symptoms after treatment; A13: Clinical efficacy (qualitative outcome); A14: RQLQ scores for emotion, nonnasal/ocular symptoms, and behavioral problem areas; A15: RQLQ sleep domain score; A16: Recurrence rate in follow-up; EA: Electric acupuncture; D: Day; W: week; M: month; Y: year; NR: nothing reported.

## Abbreviations

AR: Allergic rhinitis
SGA: Sphenopalatine ganglion acupoint
ORA: Other regular acupoints
WM: Western medicine
SA: Sham acupuncture
RR: Risk ratio
MD: Mean difference
CI: Confidence interval
TNSS: Total nasal symptom scores
TNNSS: Total nonnasal symptom scores
RQLQ: Rhinitis quality of life questionnaire.
